# Impact of obesity in the identification of the sentinel lymph node in endometrial cancer: a retrospective, monocentric study and literature review

**DOI:** 10.1007/s00404-024-07386-5

**Published:** 2024-02-24

**Authors:** Giulio Insalaco, Giosuè Giordano Incognito, Fortunato Genovese, Ferdinando Antonio Gulino, Luca Rivoli, Fabio Ciancio, Gaetano Valenti, Dalila Incognito, Ludovico Carbone, Marco Palumbo

**Affiliations:** 1 Humanitas Medical Care, Catania, Italy; 2 Department of General Surgery and Medical Surgical Specialties, University of Catania, Catania, Italy; 3 Unit of Gynecology and Obstetrics, Department of Human Pathology of Adults and Developmental Age, “G. Martino” University Hospital, Messina, Italy; 4 Medical Oncology Unit, Department of Human Pathology “G. Barresi”, University of Messina, Messina, Italy; 5 Department of Medicine, Surgery and Neurosciences, University of Siena, Siena, Italy

**Keywords:** Sentinel lymph node, Lymphadenectomy, Endometrial carcinoma, Laparoscopy, Obesity

## Abstract

**Purpose:**

To evaluate the sentinel lymph node (SLN) protocol for staging endometrial carcinomas, assessing its impact on surgical management, and determining indications for adjuvant therapies. The study also examines factors that may influence SNL mapping, particularly focusing on the failure of the technique due to obesity.

**Methods:**

A retrospective analysis was conducted on the medical records of patients with a histological diagnosis of endometrial carcinoma, who underwent surgical staging with SLN biopsy. The lymph node status was compared between non-obese (group 1) and obese (group 2) patients.

**Results:**

71 women were included in the study, of which 33 were non-obese (46.5%) and 38 were obese (53.5%). The failure detection rate was higher in obese patients (14, 36.8%) compared to non-obese patients (5, 15.2%) (*p* = 0.039). The risk of mapping failure increased by 1.6 times for every 5-unit increase in body mass index (BMI) (OR 1.672, 95% CI 1.024–2.730, *p* = 0.040). BMI was confirmed as an independent risk factor for mapping failure in both univariate (OR 3.267, 95% CI 1.027–10.395, *p* = 0.045) and multivariate analyses (OR 5.779, 95% CI 1.320–25.297, *p* = 0.020).

**Conclusion:**

SLN detection in obese patients requires great care, as obesity may alter the sensitivity of the technique.

## What does this study add to the clinical work?

**Table:** 

Despite numerous evidence supporting the high sensitivity of the sentinel lymph node biopsy technique in endometrial cancer, various factors in surgery influence mapping, including the integrity of the lymphatic drainage system Sentinel lymph node detection in obese patients requires great care, as obesity may alter the migration of the tracer and the sensitivity of the technique	

## Introduction

Endometrial carcinoma is the most common gynecological malignancy in developed countries and is almost always diagnosed in its early stages, with a five-year survival rate of 90% [1]. It can spread through direct extension, free implantation in the peritoneal cavity via the fallopian tubes, and lymphatic and hematogenous routes. Specifically, the lymphatic spread is secondary to the myometrial infiltration, allowing malignant cells to reach the parametrium, vagina, ovaries, and pelvic and lumbo-aortic lymph nodes. Beyond the International Federation of Gynecology and Obstetrics (FIGO) stage, the main prognostic factors are the cellular differentiation grade, histotype, lymphovascular space invasion (LVSI), and tumor volume [2].

The gold standard treatment is surgery, which involves radical hysterectomy [3] combined with bilateral salpingo-oophorectomy and peritoneal washing for cytological evaluation [4]. The conventional laparotomic surgical approach has given way to minimally invasive surgeries, such as laparoscopic or robotic approaches, which improve patient outcomes by significantly reducing comorbidities, postoperative complications, and hospital stays.

Many studies [5, 6] have explored the role of pelvic and/or lumbo-aortic lymphadenectomy in the management of endometrial carcinoma. They agree that it does not have therapeutic value in low-risk carcinomas, laying the groundwork for the introduction of the sentinel lymph node (SLN) protocol for staging early carcinomas and supporting the impact of this technique for surgical management and indications for adjuvant therapies.

The SLN is the first lymph node in the lymphatic chain that receives drainage from the tumor. This practice allows for the assessment of the lymph nodes' status without the need for complex lymph node dissection in patients with a low probability of lymph node metastasis, which is associated with a higher risk of postoperative complications [7]. The detection of the SLN can be achieved through various techniques. One of the most common employs is the injection of a tracer, such as indocyanine green (ICG) or blue dye, into the cervical or uterine tissue, from which the dye migrates through the lymphatic vessels to the SLN, making it visible during surgery [8]. Once identified and removed, the SLN is examined through histopathology and immunohistochemistry techniques to detect metastases. Surgical management varies depending on the outcome of the SLN analysis: the presence of tumor cells indicates that an extended lymphadenectomy should be considered, as well as in cases where the SLN is not identified and is often considered indicative of the need for adjuvant treatments such as radiotherapy or chemotherapy; in the absence of metastases, further lymph node dissection can be avoided, thus reducing surgical risks and post-operative complications [9]. Therefore, knowledge of the pelvic lymphatic pathways is essential for the correct identification of the SLN. The pelvic lymph nodes are generally the first sites of metastasis for endometrial tumors. Precise lymphatic mapping, guided by both anatomical knowledge and advanced imaging techniques, is crucial for accurate localization of the SLN [10].

Despite numerous evidence supporting the high sensitivity of the SLN biopsy technique, various factors in surgery influence mapping, thus reducing its sensitivity, including the integrity of the lymphatic drainage system. Obesity could alter the migration of the tracer, as it causes damage to the normal functioning of the lymphatic vascular system, leading to the failure of the technique. Several studies have been conducted to confirm or refute this theory [11–17].

Given the conflicting results obtained on the role of body mass index (BMI) in altering tracer uptake, this study aims to assess the extent of obesity's influence on SLN detection.

## Material and methods

This is a monocentric, retrospective analysis of the medical records of all patients admitted between January 2019 and December 2022 with a histological diagnosis of endometrial carcinoma. Regardless of the histological type, all patients who underwent surgical staging with SLN biopsy with cervical injection of ICG were enrolled.

Patients were divided into two groups based on BMI: specifically, group 1 included non-obese patients (BMI < 30 kg/m^2^), while group 2 included obese patients (BMI ≥ 30 kg/m^2^). Various variables were evaluated and compared between the two groups, including age, clinical history (previous uterine, abdominal, or pelvic surgery), and the characteristics of the surgery the patients underwent, namely whether they were subjected to omentectomy or pelvic or pelvic and lumbo-aortic lymphadenectomy. Finally, the histological characteristics of the tumors were considered and compared: histotype (endometrioid, non-endometrioid), grading, LVSI, neoplasm diameter, myometrial and cervical invasion, FIGO staging, and classification of the tumors based on the prognostic risk according to the European Society of Gynecologic Oncology/European Society of Radiation Therapy and Oncology/European Society of Pathology (ESMO-ESGO-ESP) 2020 guidelines.

Data on the lymph node status of the entire population were also evaluated by comparing the results obtained in the two groups based on BMI. Some parameters were analyzed, namely the overall detection (mono and bilateral uptake), mapping success (bilateral uptake), and failure (absent or unilateral uptake) rates. The site of uptake (above or below the ureter) and the type of findings highlighted in the histological examination of the sample, such as the presence of micro- or macrometastases, isolated tumor cells (ITC) or the identification of a negative sample were also compared. Finally, data regarding the adjuvant treatment chosen for patients were considered based on the prognostic risk and the incidence of intraoperative complications.

The primary endpoint of the study was to highlight any differences in the overall SLN detection rate, bilateral mapping, and mapping failure rate between the two population groups considered. Furthermore, another aspect evaluated was the influence of BMI, in terms of risk, on the detection rate of the SLN.

On the other hand, the secondary endpoint was to analyze the factors that could potentially affect the uptake of the SLN in the general population, both in univariate and multivariate analysis. In addition, any differences in the localization of the SLN in the population groups were studied and the histological characteristics of the histopathological findings (percentage of patients with lymph node metastases) were compared. In conclusion, the two groups, stratified based on prognostic risk, were also compared in terms of postoperative management.

The study was conducted in accordance with the Declaration of Helsinki. The institution's Ethics Committee waived the requirement of ethical approval because the study used previously archived data. Informed consent for anonymous data review and publication was obtained from all subjects involved in the study.

## Results

In total, 71 patients were enrolled, of which 33 were non-obese (46.5%) and 38 were obese (53.5%).

The clinical, surgical, and histopathological characteristics are shown in Table 1. The two groups were homogeneous for all the variables considered. In particular, they were comparable in terms of age (< 65 years (group 1 n = 19, 57.6%; group 2 n = 18, 47.4%), ≥ 65 years (group 1 n = 14, 42.4%; group 2 n = 20, 52.6%)), histotype (endometrioid (group 1 n = 29, 97.9%; group 2 n = 37, 97.4%), non-endometrioid (group 1 n = 4, 12.1%; group 2 n = 1, 2.6%)), LVSI (presence group 1 n = 9, 27.3%; group 2 n = 9, 23.7%; absence group 1 n = 24, 72.7%; group 2 n = 29, 76.3%), i.e., for the variables that could most significantly negatively affect the identification of the SLN.

**Table 1:** Clinical, surgical, and histopathological characteristics of the included population

Variables	BMI < 30 kg/m^2^ n = 33 n (%)	BMI ≥ 30 kg/m^2^ n = 38 n (%)	*p* value χ^2^
*Clinical characteristics*			
**Age**			0.390
<65 years	19 (57.6)	18 (47.4)	
≥65 years	14 (42.4)	20 (52.6)	
**Previous uterine surgery**	2 (6.1)	1 (2.6)	0.594
**Previous abdominal or pelvic surgery**	15 (45.5)	15 (39.5)	0.611
*Surgical characteristics*			
**Lymphadenectomy**			
Pelvic	8 (24.2)	14 (36.8)	0.252
Pelvic and lumbo-aortic	2 (6.1)	2 (5.3)	0.999
**Omentectomy**	4 (12.1)	4 (10.5)	
*Histopathological characteristics*			
**Histotype**			0.176
Endometrioid	29 (87.9)	37 (97.4)	
Non-endometrioid	4 (12.1)	1 (2.6)	
**Grading**			0.090
1–2	23 (69.7)	33 (86.8)	
3	10 (30.3)	5 (13.2)	
**LVSI**			0.729
No	24 (72.7)	29 (76.3)	
Yes	9 (27.3)	9 (23.7)	
**Neoplasm diameter**			0.817
<20 mm	13 (39.4)	16 (42.1)	
≥20 mm	20 (60.6)	22 (57.9)	
**Myometrial invasion**			0.651
<50%	20 (60.6)	25 (65.8)	
≥ 50%	13 (39.4)	13 (34.2)	
**Cervical invasion**			0.671
No	21 (63.6)	26 (68.4)	
Yes	12 (36.4)	12 (31.6)	
**FIGO staging**			0.520
IA	14 (42.4)	19 (50.0)	
IB	2 (6.1)	4 (10.5)	
II	9 (27.3)	10 (26.3)	
IIIA–IIIB	2 (6.1)	0 (0.0)	
IIIC	6 (18.2)	5 (13.2)	
**Classification based on prognostic risk**			0.130
Low	12 (36.4)	17 (44.7)	
Intermediate	1 (3.0)	5 (13.2)	
High-intermediate	9 (27.3)	11 (28.9)	
High	11 (33.3)	5 (13.2)	
Advanced metastatic			

*BMI* body mass index, *LVSI* lymphovascular space invasion, *n* number

Table 2 reports the data on the lymph node status of the entire population, stratified into two groups based on BMI. The overall detection rate was homogeneous in the two groups; however, both the mapping success and the failure rates showed statistically significant differences between the two groups (*p* = 0.039). Specifically, the failure rate was 15.2% in patients with BMI < 30 kg/m^2^, while in the group of obese patients, the success rate was 36.8%. Although this data did not reach statistical significance, it is interesting to note that, due to comorbidities, surgical difficulties, or anesthesiological challenges encountered, 4 patients in the second group (10.5%) were not staged compared to 0 patients in the first group. The localization of the SLN (supra/infra-ureteral route) was found to be comparable between the two groups. Finally, the histopathological findings in the SLN were also homogeneous: negative lymph nodes were found in 80.6% and 82.4% of the cases in the patient groups with BMI < 30 kg/m^2^ and BMI ≥ 30 kg/m^2^, respectively; ITC were found in a single lymph node in both groups; the number of micrometastases found was 3 in the non-obese patient group and 0 in the obese patient group; finally, the number of macrometastases identified was 2 in the first group and 5 in the second group.

**Table 2:** Lymph node status of the entire population stratified into groups based on BMI

Variables	BMI < 30 kg/m^2^ n = 33 n (%)	BMI ≥ 30 kg/m^2^ n = 38 n (%)	*p* value χ2
**Overall detection rate (mono-bilateral)**	31 (93.9)	33 (86.8)	0.438
**Mapping success (bilateral)**	28 (84.8)	24 (63.2)	**0.039**
**Mapping failure (no/monolateral)**	5 (15.2)	14 (36.8)	
**Non-classified patients (due to comorbidities and no mapping)**	0 (0.0)	4 (10.5)	0.118
**Right hemipelvis**			0.491
Supra-ureteral pathway	28 (100)	27 (93.1)	
Sub-ureteral pathway	0 (0.0)	2 (6.9)	
**Left hemipelvis**			0.599
Supra-ureteral pathway	30 (96.8)	26 (92.9)	
Sub-ureteral pathway	1 (3.2)	2 (7.1)	
**Histopathological findings in SLN (overall detection)**			0.859
**Negative**			
Negative	25 (80.6)	28 (82.4)	
**Positive**	6 (19.4)	6 (17.6)	
ITC	1	1	
Micrometastasis	3	0	
Macrometastasis	2	5	

*BMI* body mass index, *n* number, *ITC* isolated tumor cells, *SLN* sentinel lymph node

**Fig. 1:**
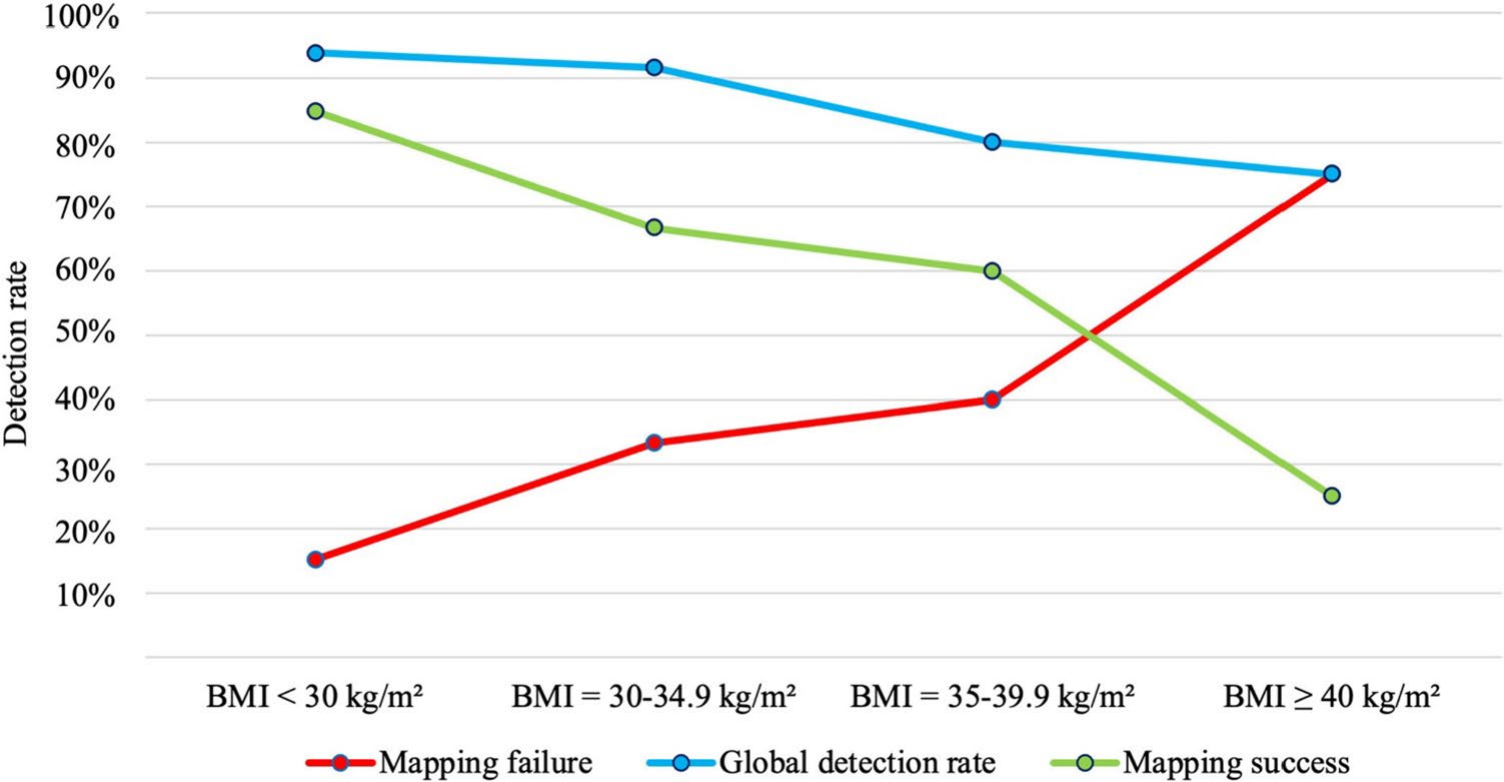
Binary logistic regression analysis per 5-unit increase in BMI. Univariate analysis per 5 kg/m^2^ increment. Mapping failure risk: OR 1.672, 95% CI 1.024–2.730, *p* = 0.040. Successful mapping rate: OR 0.598, 95% CI 0.366–0.976, *p* = 0.040. Overall detection rate: OR 0.665, 95% CI 0.333–1.325, *p* = 0.246

In the binary logistic regression, lymph node uptake was strongly influenced by BMI. Specifically, it was observed that the risk of mapping failure increased by 1.6 times for every 5-unit increase in BMI (odds ratio, OR 1.672, 95% confidence interval, CI 1.024–2.730, *p* = 0.040), resulting in a statistically significant decrease in the probability of success (OR 0.598, 95% CI 0.366–0.976, *p* = 0.040). The overall detection rate also decreased with increasing BMI, although this result did not reach statistical significance. Indeed, from 93.9% detected in the group of patients with BMI < 30 kg/m^2^, it dropped to 75% in patients with BMI ≥ 30 kg/m^2^ (OR 0.665, 95% CI 0.333–1.325, *p* = 0.246) (Fig. 1, Table 3).

**Table 3:** Frequency table

BMI (kg/m^2^)	n	Global detection (mono-bilateral) n (%)	Mapping failure (missed/monolateral) n (%)	Mapping success (bilateral) n (%)
<30	33	31 (93.9)	5 (15.2)	28 (84.8)
30–34.9	24	22 (91.6)	8 (33.3)	16 (66.7)
35–39.9	10	8 (80.0)	4 (40.0)	6 (60.0)
≥40	4	3 (75.0)	3 (75.0)	1 (25.0)

*BMI* body mass index, *n* number

As reported in Table 4, only BMI has been confirmed as a risk factor for SNL mapping failure in the univariate analysis (OR 3.267, 95% CI 1.027–10.395, *p* = 0.045). This data was also confirmed in the multivariate analysis (OR 5.779, 95% CI 1.320–25.297, *p* = 0.020).

**Table 4:** Uni- and multivariate analysis on the mapping failure

Variables	Univariate analysis		Multivariate analysis	
	OR (95% CI)	*p* value	OR (95% CI)	*p* value
**Age**		0.124		0.131
<65 years	Reference		Reference	
≥65 years	2.338 (0.792–6.900)		2.696 (0.745–9.750)	
**Previous uterine surgery**		0.794		0.675
None	Reference		Reference	
Yes	1.389 (0.119–16.259)		0.481 (0.016–14.632)	
**BMI**		0.045		0.020
<30 kg/m^2^	Reference		Reference	
≥30 kg/m^2^	3.267 (1.027–10.395)		5.779 (1.320–25.297)	
**Histotype**		0.106		0.163
Endometrioid	Reference		Reference	
Non-endometrioid	4.487 (0.718–30–587)		7.978 (0.432–147–196)	
**Grading**		0.198		0.442
1–2	Reference		Reference	
3	2.205 (0.661–7.358)		2.060 (0.327–12.990)	
**LVSI**		0.616		0.939
No	Reference		Reference	
Yes	0.724 (0.205–2.2556)		1.091 (0.118–10.107)	
**Neoplasm diameter**		0.340		0.196
<20 mm	Reference		Reference	
≥20 mm	1.718 (0.566–5.222)		2.521 (0.620–10.255)	
**Myometrial invasion**		0.280		0.242
<50%	Reference		Reference	
≥50%	0.527 (0.165–1.685)		0.242 (0.036–1.648)	
**Cervical invasion**		0.744		0.879
No	Reference		Reference	
Yes	1.201 (0.401–3.599)		0.889 (0.194–4.059)	

*BMI* body mass index, *CI* confidence interval, *LVSI* lymphovascular space invasion, *OR* odds ratio

Table 5 shows the intraoperative complications and adjuvant treatments to which each of the two groups was subjected. Few intraoperative complications were reported, and no statistically significant difference was found between the groups. The decision on adjuvant treatment was based on the prognostic risk of the patients, divided into four different risk groups, as recommended by the ESMO-ESGO-ESP 2021 guidelines; however, no statistically significant differences emerged between patients with a BMI ≥ 30 kg/m^2^ and those with a BMI < 30 kg/m^2^ among the different prognostic groups.

**Table 5:** Intraoperative complications and adjuvant treatments

Variables	BMI < 30 kg/m^2^ n = 33 n (%)	BMI ≥ 30 kg/m^2^ n = 38 n (%)	*p* value χ2
**EBL, mL (mean, range)**	50 (0–100)	50 (50–200)	0.290
**Intraoperative complications**	1 (3.0)	1 (2.6)	0.999
Visceral injuries	1	0	
Vascular injuries	0	1	
*Adjuvant treatment based on prognostic risk*			
**Low**	12	17	0.999
FUP	12 (100)	17 (100)	
**Intermediate**	1	5	
FUP	0	0	
EBRT/BRT	1 (100)	5 (100)	
**High-intermediate**	9	11	0.350
FUP	0 (0.0)	2 (18.2)	
EBRT/BRT	6 (66.7)	7 (63.6)	
CHT + RT	3 (33.3)	2 (18.2)	
**High**	11	5	0.486
FUP	1 (9.1)	0 (0.0)	
CHT + RT	10 (90.9)	5 (100)	

*BRT* brachytherapy, *CHT* chemotherapy, *EBL* estimated blood loss, *EBRT* external beam radiation therapy, *FUP* follow-up, *RT* radiotherapy

## Discussion

First included in the National Comprehensive Cancer Network (NCCN) 2014 guidelines as a staging surgical option for neoplasia confined to the uterus, the SLN protocol is now established as a technique equivalent to lymphadenectomy in early-stage endometrial carcinoma [12], with the advantage of selectively removing the lymph node that directly drains from the primary tumor site after dye injection, avoiding the comorbidities of an extended lymphadenectomy [18].

Many authors have tried to identify factors (patient characteristics, operator limitations, lymphatic vessel obstruction) that affect mapping, thereby reducing its sensitivity and ensuring the best therapeutic strategy for patients. Indeed, if predictive factors for mapping failure could be identified early, strategies could be developed to increase the success rate of mapping even in these cases. Some have assessed the role of obesity in altering tracer uptake, but the results have been contradictory [11–17] (Table 6). Tanner et al. [11] and Sinno et al. [12] identified a statistically significant difference between the mapping success rates in obese and non-obese patients; however, they concluded that using ICG instead of blu dye could overcome the negative effect of obesity on mapping. A retrospective analysis by Taşkın et al. [13] supported this evidence, asserting that no significant difference in mapping success rates was found using ICG as a dye between obese and non-obese patients. Another study by Ianieri et al. [14] further explored the role of the integrity of the lymphatic system, concluding that neither lymph node metastases nor obesity were significantly associated with an increased rate of mapping failure. On the contrary, the only factor proven to reduce the success rate was the skill of the surgeon performing the technique. Contrary to what other studies suggest, Eriksson et al. [15], while confirming the superiority of ICG over methylene blue, also noted that the mapping success rate gradually decreased as BMI increased with both tracers. Similarly, a trial by Eitan et al. [16] reached the same conclusion, that a higher BMI was associated with mapping failure in multivariate analysis. However, this study also noted that LVSI and the surgeon's experience could alter the mapping outcome for endometrial carcinoma. Finally, the most recent study conducted in 2020 by Sozzi et al. [17], aiming to identify factors associated with SLN mapping failure in patients with endometrial carcinoma, concluded that BMI was not a statistically significant factor in reducing the mapping success rate, thus contradicting previous evidence. Instead, a non-endometrioid histotype, LVSI, and evidence of enlarged lymph nodes were found to be independent risk factors for unsuccessful lymph node mapping.

**Table 6:** Studies assessing the impact of obesity in the SNL identification in endometrial cancer

References	Country	Study size (n)	Study method	Time period	Tracer used	Injection site	Route of surgery	Results
Tanner et al. [11]	United States	111 (non-obese: 47; obese: 64)	Prospective	September 2012–November 2014	ICG and blue dye	Cervical	Robotic	Negative association between BMI and bilateral mapping success in both univariate (OR 0.96, 95% CI 0.92–1.00) and multivariate analysis (OR 0.95, 95% CI 0.91–0.99). When stratified by dye type, the association was only significant for blue dye (OR 0.92, 95% CI 0.84–0.99) and not for ICG
Sinno et al. [12]	United States	71	Prospective	September 2012–March 2014	ICG and blue dye	Cervical	Robotic	Negative association between BMI and bilateral mapping success in both univariate OR 0.9, 95% CI 0.88–0.99, *p* = 0.043) and multivariate analysis (OR 0.91, 95% CI 0.85–0.98, *p* = 0.022). When stratified by dye type, the association was only significant for blue dye (*p* = 0.03) and not for ICG (*p* = 0.14)
Taşkın et al. [13]	Turkey	88 (non-obese: 41; obese: 47)	Retrospective	April 2017–October 2018	ICG	Cervical	Laparoscopy, laparotomy, and robotic	No association between BMI and SNL mapping (*p* = 0.87)
Ianieri et al. [14]	Italy	110	Prospective	June 2017–June 2018	ICG	Cervical	Laparoscopy	No association between BMI and SNL mapping (*p* = 0.578)
Eriksson et al. [15]	United States	472 (non-obese: 222; obese: 250)	Retrospective	2011–2013	ICG and blue dye	Cervical	Robotic	Negative association between BMI and bilateral mapping success for both ICG (p < 0.001) and blue dye groups (*p* = 0.041). The use of ICG resulted in better bilateral (*p* = 0.002) and overall (*p* = 0.011) mapping rates compared with the use of blue dye in all BMI groups
Eitan et al. [16]	Israel	74	Prospective	January 2012–December 2014	Blue dye	Cervical	Robotic	Negative association between BMI and bilateral mapping success in multivariate analysis (OR = 0.899, 95% CI 0.808–1.00)
Sozzi et al. [17]	Italy	376	Retrospective	January 2016–July 2019	ICG	Cervical	Laparoscopy	No association between BMI and SNL mapping (OR 1, 95% CI 0.96–1.03, *p* = 0.98)

*BMI* body mass index, *CI* confidence interval, *ICG* indocyanine green, *n* number, *OR* odds ratio, *SNL* sentinel lymph node

Considering the present study, BMI was confirmed as an independent risk factor for the failure of the SLN technique, first in univariate analysis and then in multivariate analysis. Indeed, in the group of patients with BMI ≥ 30 kg/m^2^, a statistically higher rate of mapping failure was observed compared to the group with BMI < 30 kg/m^2^, with a failure rate of 36.8% in the first group compared to 15.2% in the second, and with a p-value of 0.039. Furthermore, after performing a binary logistic regression, the detection rate was found to decrease for every 5 units of BMI increase, with statistically significant values. Finally, it is interesting to note that, although not statistically significant, 10.5% of obese patients were not staged. In these patients, the SLN algorithm was not applied due to problems related to the management of the surgery, either by the decision of the surgeon or due to anesthesiological or surgical complications. In contrast, no cases of understaging were observed among the non-obese patient group. This finding could be considered particularly relevant since the percentages of lymph node metastases detected in the two groups were comparable. This means that obese patients risk not being directed towards the correct postoperative therapeutic pathway, as understaging inevitably results in exclusion from adjuvant treatment. Therefore, the authors believe that SLN detection should be performed with great care in obese patients, considering the difficulties caused by obesity itself. All this to avoid lymphadenectomy and simultaneously achieve the best possible result from the SLN biopsy. Additionally, a low incidence of intraoperative complications was noted, which was comparable between the two groups. This further underscores that understaging in obese patients is not justified.

One of the strengths of the study lies in the several factors assessed that could influence the success of SLN mapping, focusing particularly on the role of BMI. This nuanced approach provides a well-rounded understanding of the complexities involved in SLN mapping for endometrial carcinoma. Another notable aspect is the rigorous statistical methodology, employing both univariate and multivariate analyses to ascertain the influence of BMI on the SLN technique's failure rates. However, the study is not without its limitations. One of the key challenges faced in this research is the variability in surgical skills, which could significantly influence the outcome of SLN mapping. Furthermore, its findings are limited to a specific patient population and set of clinical conditions. This specificity means that the results may not be universally applicable, and care should be taken when extrapolating these findings to broader patient groups or different clinical settings. Further prospective studies are needed to confirm these results and to explore the underlying mechanisms in detail.

## Data Availability

Not applicable.
